# Detection of Leptomeningeal Metastasis by Contrast-Enhanced 3D T1-SPACE: Comparison with 2D FLAIR and Contrast-Enhanced 2D T1-Weighted Images

**DOI:** 10.1371/journal.pone.0163081

**Published:** 2016-10-03

**Authors:** Bomi Gil, Eo-Jin Hwang, Song Lee, Jinhee Jang, Hyun Seok Choi, So-Lyung Jung, Kook-Jin Ahn, Bum-soo Kim

**Affiliations:** Departments of Radiology Seoul St. Mary’s Hospital, College of Medicine, The Catholic University of Korea, Seoul, South Korea; University of Michigan, UNITED STATES

## Abstract

**Introduction:**

To compare the diagnostic accuracy of contrast-enhanced 3D(dimensional) T1-weighted sampling perfection with application-optimized contrasts by using different flip angle evolutions (T1-SPACE), 2D fluid attenuated inversion recovery (FLAIR) images and 2D contrast-enhanced T1-weighted image in detection of leptomeningeal metastasis except for invasive procedures such as a CSF tapping.

**Materials and Methods:**

Three groups of patients were included retrospectively for 9 months (from 2013-04-01 to 2013-12-31). Group 1 patients with positive malignant cells in CSF cytology (n = 22); group 2, stroke patients with steno-occlusion in ICA or MCA (n = 16); and group 3, patients with negative results on MRI, whose symptom were dizziness or headache (n = 25). A total of 63 sets of MR images are separately collected and randomly arranged: (1) CE 3D T1-SPACE; (2) 2D FLAIR; and (3) CE T1-GRE using a 3-Tesla MR system. A faculty neuroradiologist with 8-year-experience and another 2^nd^ grade trainee in radiology reviewed each MR image- blinded by the results of CSF cytology and coded their observations as positives or negatives of leptomeningeal metastasis. The CSF cytology result was considered as a gold standard. Sensitivity and specificity of each MR images were calculated. Diagnostic accuracy was compared using a McNemar’s test. A Cohen's kappa analysis was performed to assess inter-observer agreements.

**Results:**

Diagnostic accuracy was not different between 3D T1-SPACE and CSF cytology by both raters. However, the accuracy test of 2D FLAIR and 2D contrast-enhanced T1-weighted GRE was inconsistent by the two raters. The Kappa statistic results were 0.657 (3D T1-SPACE), 0.420 (2D FLAIR), and 0.160 (2D contrast-enhanced T1-weighted GRE). The 3D T1-SPACE images showed the highest inter-observer agreements between the raters.

**Conclusions:**

Compared to 2D FLAIR and 2D contrast-enhanced T1-weighted GRE, contrast-enhanced 3D T1 SPACE showed a better detection rate of leptomeningeal metastasis.

## Introduction

Brain metastases are the most common intracranial tumors in adults, affecting 20% of the patients with cancer [1]. Parenchymal metastasis is discovered by a bright nodular necrotic mass on a contrast enhanced T1-weighted MRI [2]. This phenomenon is explained by contrast agent leakage from tumor vessels, which is due to the disorganized blood-brain barriers. Therefore, parenchymal metastasis shows a good contrast to normal brain parenchyma. A gadolinium-enhanced MRI is preferred for an initial evaluation of cancer staging due to its minimal invasiveness [3,4]. However, leptomeningeal metastasis is often missed on MRI because leptomeninx is anatomically such a thin membrane that subtle enhancement can be ignored by readers. Leptomeningeal metastasis can also be detected by fluid attenuated inversion recovery (FLAIR) images. Sulcal hyperintensities on FLAIR are not specific for leptomeningeal metastasis, and they are also associated with various conditions such as subarachnoid hemorrhage, sluggish collateral vessels, and supplemental oxygen. Therefore, FLAIR images may produce false positive interpretations on leptomeningeal metastasis. Because leptomeningeal vessels can show enhancement on the contrast-enhanced (CE) T1-weighted gradient echo images (GRE), leptomeningeal metastasis can be misinterpreted as leptomeningeal vessels. This is a presumptive cause of false negative interpretation of leptomeningeal metastasis. An accurate detection of leptomeningeal metastasis on MRI is important for determining initial cancer staging and for treatment plannings. Cytopathology of cerebrospinal fluid (CSF) has been considered as a gold standard to confirm leptomeningeal metastasis, but its sensitivity is not as strong as it is expected [5,6].

A recently introduced T1-weighted sampling perfection with application-optimized contrasts by using different flip angle evolutions (T1-SPACE) is one of the 3D spin echo sequences, which nullifies signals from moving flows. Higher lesion detectability can be achieved by a better contrast-to-noise ratio and lesion clarity with less false positives compared with other gradient echo sequences [7]. We hypothesized that CE T1-SPACE has a better diagnostic performance on detecting leptomeningeal metastasis compared with FLAIR and CE gradient echo T1-weighted images. This study was aimed to compare the diagnostic accuracy of 3D CE T1-SPACE, 2D FLAIR and 2D CE T1-weighted images in detection of leptomeningeal metastasis.

## Materials and Methods

### Patients and subjects

This retrospective study was approved and waived for patients’ consents by the institutional review board (IRB). Between April 2013 and December 2013, 134 patients with positive malignant cells, who completed CSF tapping, were included in this study. Among these patients, 22 patients underwent brain MRI with 2D FLAIR, 3D CE T1 SPACE and 2D CE T1 GRE. These patients were considered as a case group. Sixteen acute stroke patients with MCA occlusion were considered as a control group A. Twenty five patients with complaint of dizziness, headache, and syncope, who were diagnosed to be negative on MR images, were classified as a control group B. Both control group A and B underwent 2D FLAIR, 3D T1 CE SPACE and 2D CE T1 GRE.

### Acquisition of MRI

All images were obtained using a 3T MR system (Verio, Siemens Medical Solutions, Erlangen, Germany). The 2D FLAIR sequence was performed with the following parameters: repetition time and echo time (TR/TE) = 9000/96 msec; inversion time of 2500 msec; 5-mm slice thickness, 1-mm inter-slice gap, 220-mm of field of view; 384x288 matrix size; and acquisition time of 2 minutes and 26 seconds. The contrast-enhanced 2D T1 GRE sequence was obtained 3 minutes after the intra-venous injection of 0.1 mmol/kg of gadobutrol (Gadovist, Bayer Healthcare, Germany) at a rate of 1.5 mL/s with the following parameters: repetition time and echo time (TR/TE) = 250/3 msec; 5-mm slice thickness, 1-mm inter-slice gap, 220-mm of field of view; 448x336 matrix size; and acquisition time of 1 minute and 44 seconds. The contrast-enhanced 3D T1 SPACE sequence was subsequently acquired after obtaining the 2D T1 GRE sequence: repetition time and echo time (TR/TE) = 700/11 msec; 1-mm slice thickness, no inter-slice gap, 250-mm of field of view; 256x256 matrix size; and acquisition time of 4 minutes 23 seconds.

### Image analysis

A total of 63 sets of 2D FLAIR, 3D CE T1 SPACE and 2D CE T1 GRE were collected and randomly distributed in order by a 6-year experienced neuroradiologist (JHJ). Two raters analyzed 63 sets of 2D FLAIR, 3D CE T1 SPACE and 2D CE T1 GRE to determine whether the images were positive of leptomeningeal metastasis or not. They were different in levels of experiences: an 8-year experienced faculty neuroradiologist (HSC) and a second-year radiology trainee (BMG). The positive of leptomeningeal metastasis was defined by high leptomeningeal signals on 2D FLAIR, 3D CE T1 SPACE, or on 2D CE GRE. Floating linear high signal separated by the brain surfaces on 2D FLAIR, 3D CE T1 SPACE, and 2D CE GRE were regarded as leptomeningeal collateral vessels, negative of leptomeningeal metastasis. The raters were blinded by the order of different MR sequences and by the results of the CSF cytology. The CSF cytology result was considered as a gold standard for detecting leptomeningeal metastasis, and a 2X2 contingency table was for analysis of the diagnostic performances.

### Statistical analysis

Sensitivity, specificity, positive predictive values, and negative predictive values of the different MR sequences by the two raters were calculated. A comparison of diagnostic accuracy of leptomeningeal metastasis among the different MR sequences and of the CSF cytology results were performed using a McNemar’s test. Cohen's kappa was calculated to evaluate inter-observer agreements.

## Results

### Diagnostic performance of the different MR sequences

**Fig 1:**
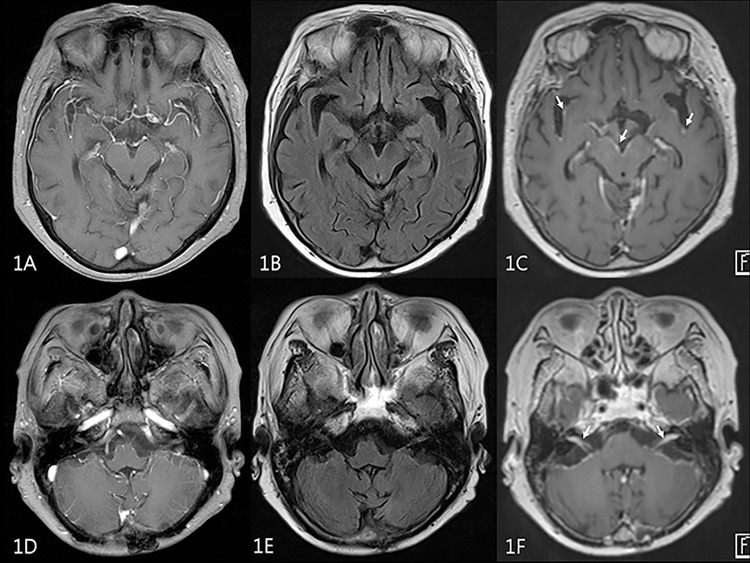
MR images of 72 year-old female patient with breast cancer. 2D contrast-enhanced T1-weighted GRE (A,D) and 2D FLAIR (B,E) were negatively interpreted by a trainee rater. On 3D contrast-enhanced T1-SPACE (C,F), leptomeningeal enhancement along interpeduncular cistern, sylvian fissures (arrows on 1C) and internal auditory canals (arrows on 1F) was seen.

**Fig 2:**
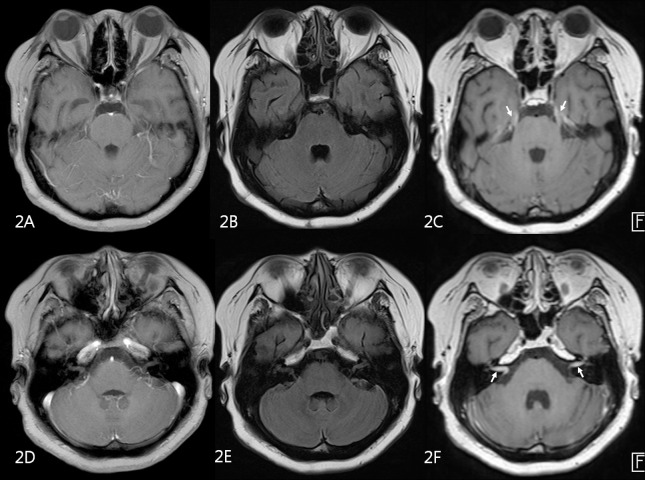
MR images of 58 year-old male patient with advanced gastric cancer. 2D contrast-enhanced T1-weighted GRE (A,D) and 2D FLAIR (B,E) were negatively interpreted by both faculty and trainee raters. On 3D contrast-enhanced T1-SPACE (C,F), leptomeningeal enhancement along trigeminal nerves (arrows on C) and internal auditory canals (arrows on F) was seen.

The sensitivity and specificity of leptomeningeal metastasis by the 8-year experienced faculty neuroradiologist were 81.82% and 95.12% (3D CE T1-SPACE); 68.18% and 90.24% (2D FLAIR); and 54.55% and 92.68% (2D CE T1-GRE), respectively. The sensitivity and specificity by the second-year trainee radiologist were 72.73% and 97.56% (3D CE T1-SPACE); 45.45% and 92.68% (2D FLAIR); and 36.36% and 95.12% (2D CE T1- GRE), respectively. The Faculty’s rating showed a higher sensitivity and a lower specificity, but there was an overlap of 95% confidence interval between the two raters (Tables 1 and 2). 3D CE T1-SPACE depicted subtle leptomeningeal enhancement compared with 2D FLAIR and 2D CE T1-GRE regardless of faculty and trainee raters (Figs 1 and 2).

**Table 1:** Diagnostic performance of different sequences by Reader 1.

MR sequences	TP	TN	FP	FN	Sensitivity(%)	Specificity(%)	PPV (%)	NPV (%)
**3D CE T1-SPACE**	18	39	2	4	81.82(58.99–94.01)	95.12(82.19–99.15)	90.00(66.87–98.25)	90.70(76.95–96.98)
**2D FLAIR**	15	37	4	7	68.18(45.12–85.27)	90.24(75.94–96.83)	78.95(53.90–93.03)	84.09(69.33–92.84)
**2D CE T1-GRE**	12	38	3	10	54.55(32.67–74.93)	92.68(78.99–98.09)	80.00(51.37–94.69)	79.17(64.60–89.04)

TP refers to true positive; TN, true negative; FP, false positive; and FN, false negative. CE refers to contrast-enhanced. 95% confidence intervals were expressed within parenthesis.

**Table 2:** Diagnostic performance of different sequences by Reader 2.

MR sequences	TP	TN	FP	FN	Sensitivity(%)	Specificity(%)	PPV (%)	NPV (%)
**3D CE T1-SPACE**	16	40	1	6	72.73(49.56–88.39)	97.56(85.59–99.87)	94.12(69.24–99.69)	86.96(73.05–94.58)
**2D FLAIR**	10	38	3	12	45.45(24.39–67.7)	92.68(80.08–98.46)	76.92(46.19–94.96)	76.00(61.83–86.94)
**2D CE T1-GRE**	8	39	2	14	36.36(18.03–59.17)	95.12(82.19–99.15)	80.00(44.22–96.46)	73.58(59.42–84.32)

TP refers to true positive; TN, true negative; FP, false positive; and FN, false negative. CE refers to contrast-enhanced. 95% confidence intervals were expressed within parenthesis.

A McNemar’s test to compare diagnostic accuracy among the different MR sequences and the CSF cytology results (gold standard) showed different diagnostic accuracies in both 2D FLAIR and 2D CE T1-GRE by the trainee radiologist. However, neither 3D T1-SPACE rated by the faculty neuroradiologist nor by the trainee radiologist showed any diagnostic differences (Table 3).

**Table 3:** Results of McNemar test.

MR sequences	Reader 1	Reader 2
3D CE T1-SPACE	0.125	0.687
2D FLAIR	0.035*	0.549
2D CE T1-GRE	0.004*	0.092

CE refers to contrast-enhanced. *P-value less than 0.05.

The inter-observer agreements measured by a Cohen's kappa value were 0.657 (3D CE T1-SPACE), 0.420 (2D FLAIR), and 0.160 (2D CE T1-GRE). The 3D CE T1-SPACE images showed the highest inter-observer agreement of 0.657, which is a substantial agreement (Table 4). The 2D FLAIR showed the kappa value of 0.420 (moderate agreement), while 2D CE T1-GRE showed 0.160 (slight agreement).

**Table 4:** Degree of agreement between Reader 1 and Reader 2.

MR sequences	Cohen's kappa
3D CE T1-SPACE	0.657*
2D FLAIR	0.420
2D CE T1-weighted GRE	0.160

CE refers to contrast-enhanced. * Cohen's kappa>0.6 refers to substantial agreement.

## Discussion

The Cerebrospinal fluid (CSF) cytology has been considered as a gold standard for leptomeningeal metastasis, but its technique is invasive and its sensitivity is relatively low. Accurate diagnosis using both MRI and the CSF cytology can guide a proper treatment. For an image based approach to diagnose brain metastasis, 2D contrast-enhanced T1-weighted MRI and FLAIR have been widely used to date [3,4,8–11]. Recently, 3D FLAIR imaging has been applied to evaluate anatomy and pathology [12–14]. The 3D T1-SPACE has an advantage- of high signal-to-noise ratio due to its innate 3D spin echo based feature. Low SAR (specific absorption rate), high contrast enhancement effect, multi-planar evaluation, and black blood phenomena enable T1-SPACE a better non-invasive diagnostic modality for diagnosing brain metastasis [15,16]

A few studies compared different MR sequences to detect leptomeningeal enhancement. In the past, the sensitivity of leptomeningeal enhancement using MRI varied from 20 to 71% [3,8,17]. Ronnie and et al tested patients with clinical symptoms suspicious for leptomeningeal metastasis and reported a neuroimaging (CT or MRI) abnormality of 70 out of 128 (54.7%) [4]. Singh and et al compared the sensitivity of 2D FLAIR and CE T1-weighted spin-echo in patients with cytologically confirmed leptomeningeal metastasis: 34% in FLAIR and 66% in CE T1-weighted spin-echo [10] A subsequent study by Singh and et al showed that the sensitivity and specificity of CE FLAIR for detecting leptomeningeal metastasis were 41% and 88%, while those of CE T1-weighted MRI were 59% and 93% respectively [11]. Our results showed a comparable accuracy of 2D FLAIR and 2D CE T1-weighted GRE. 3D CE T1-SPACE showed a higher sensitivity and specificity. However, the range of accuracy varied depending on MR sequences and raters’ experiences. The sensitivity and specificity of detecting leptomeningeal metastasis by the 8-year experienced faculty neuroradiologist were 81.82% and 95.12% (3D CE T1-SPACE), while those by the second-year trainee radiologist were 45.45% and 92.68% (2D FLAIR). Further studies with larger population are needed.

A contrast-enhanced T1 SPACE showed a better detection of small parenchymal metastasis than MP-RAGE and 2D T1-weighted spin echo sequences did [18,19]. It has theoretically been supported by Mugler and Brookeman that because lesions in MP-RAGE sequences are less enhanced by gadolinium than in spin echo based sequences, lesion enhancement is likely to be missed in MP-RAGE [20]. According to Tomohiro and Shinji et al who compared the various 3T MR sequences, 3D-SPACE was the most accurate sequence to detect the brain parenchymal tumors due to its vessel signal suppression techniques [15]. In our series, vessel signal suppression may also have helped reduce some false positive interpretations, as the images from 3D CE T1-SPACE showed a smaller number of false positive cases compared with those from 2D FLAIR and 2D CE T1-GRE. Although the leptomeningeal collateral vessels in patient with acute stroke can mimic leptomeningeal metastasis, the pattern of floating curvilinear enhancement was different from the metastasis.

In our study, the diagnostic accuracy of the faculty rater on leptomeningeal metastasis was not different among the different MR sequences and the CSF cytology results. However, the diagnostic accuracy of the trainee rater was different both between 2D FLAIR and CSF cytology and between 2D CE T1-GRE and CSF cytology. The 3D CE T1-SPACE was the only consistent sequence for detection of leptomeningeal metastasis regardless of the degree of the rater’s experiences. Therefore, 3D CE T1-SPACE can be generally used for screening of leptomeningeal metastasis regardless of the reader’s experience. However, the resolution of the three sequences compared in this study was different. The 3D T1 SPACE had an inferior in-plane resolution but smaller voxel sizes compared with 2D FLAIR and 2D T1 GRE. Actual voxel sizes of the three were 0.6x0.8x5-mm in 2D FLAIR; 0.5x0.7x5-mm in 2D CE GRE; and 1x1x1-mm in 3D CE T1-SPACE. Our study had following limitations. First, the study design was retrospective, single center based, and the sample size was small. The sample size was small for detecting leptomeningeal metastasis (n = 22) because clinical diagnosis of leptomeningeal metastasis without confirmation of CSF cytology should be excluded. Although there were more cases with clinically suggestive of leptomeningeal metastasis, only the cases of leptomeningeal metastasis confirmed by the CSF cytology were included in this study. Therefore, a small number of patients with leptomeningeal metastasis were inevitable due to the restrictive inclusion criteria. A further study with a larger number of cases should be performed. Second, only 2D CE T1-GRE was used to be compared with 3D CE T1-SPACE, but the-spin echo-based 2D CE T1-weighted image should also be considered in the future. Third, raters were only answered whether there was leptomeningeal metastasis or not. This was different from clinical settings and could be a potential bias. Fourth, this study only included patients with leptomeningeal metastasis, and any other leptomeningeal pathologies other than leptomeningeal metastasis were not evaluated on this study. Finally, this study was a cross-sectional comparison for diagnostic accuracy. The effect of diagnostic accuracy on patients’ clinical outcome could not be evaluated. Further studies on early accurate detection and proper treatment in leptomeningeal metastasis should be performed to evaluate whether the accurate diagnosis induces a positive influence on patients’ lives.

## Conclusions

3D CE T1-SPACE showed the most consistent diagnostic performance with CSF cytology and the highest agreements among the faculty and trainee raters in detecting leptomeningeal metastasis.

## Supporting Information

S1 File Supporting data. Raw results of statistical analysis.
(XLS)
